# Application of the random material point method to 3D slope failures

**DOI:** 10.1007/s11440-025-02542-w

**Published:** 2025-03-20

**Authors:** Guido Remmerswaal, Philip J. Vardon, Michael A. Hicks

**Affiliations:** 1 Faculty of Civil Engineering and Geosciences, Delft University of Technology, Delft, The Netherlands; 2 Energy and Underground Infrastructure, Deltares, Delft, The Netherlands

**Keywords:** Large deformations, Random material point method (RMPM), Sensitive clays, Three-dimensional (3D) slope failure

## Abstract

Three-dimensional and spatial variability effects on slope failure processes are investigated for an idealised slope stability problem with the random material point method (RMPM). A 45 degree slope is brought to failure by either its own weight or by a combination of its own weight and an additional surface load applied at the crest. The ultimate failure load and potential failure processes are studied for various (heterogeneous) material strength profiles. In 3D, failures tend to spread sideways and backwards. For the slope geometry considered, the resistance to initial and secondary failures in 3D simulations tends to be higher than in 2D simulations, probably due to the additional resistance from the ends of the failure surfaces. The failure behaviour changes when a depth trend in the material strength is introduced. A depth trend in the material strength triggers a flow-like failure process, instead of distinct (approximately) circular failure surfaces which are encountered in a material without a depth trend. The flow-like behaviour causes an expansion in the failure zone in all directions while avoiding (where possible) local strong zones.

## Introduction

Landslides and slope failures can have drastic consequences. Large landslides, as seen for example in sensitive clays, pose a serious risk to human life [25]. Moreover, the (financial and environmental) damage caused by these landslides can affect many more people. Similar to landslides, dyke slope failures pose a large risk to human life, as they may be a trigger for flooding of the hinterland [3, 22].

Landslides are complex physical systems often comprising several stages [14]. The large variety of landslides have been categorised; see, for example, the (modified) Varnes classification [2, 14, 32] to assist in identifying other landslides exhibiting similar phenomena and characteristics. Cruden and Varnes [2] acknowledged the difficulty in categorising landslides due to the different stages in their evolution and proposed to classify each stage during a detailed investigation. Hungr et al. [14] proposed to categorise a landslide based on the stage the researcher focuses on. In (sensitive) clay landslides, four main types of failure have been observed: rotational slides, multiple retrogressive rotational slides, translational (progressive) slides, and spreads [18].

Recently, efforts have been made to reduce the risk of landslides and slope failures to an acceptable level. To assess the risk, both the likelihood and consequence of these hazards must be known. Advances in large deformation modelling, such as the material point method (MPM) [24], enable the assessment of the probability and consequence of landslides and slope failures within a single tool [5, 23, 27, 33, 35–37].

In MPM, the continuum is discretised into material points, while a background mesh is used as a computational grid. The material points can move through the background mesh [24], thereby allowing the modelling of the entire dynamic process, and for slope failure this means from failure initiation until the failure process is complete. MPM has been shown to be a useful tool in modelling the likelihood and consequence of slope failures and landslides. However, these analyses are usually performed in 2D, as indicated by Tran et al. [26], and they usually ignore the effect of spatial variability of soil properties on the failure process [21].

It is widely accepted that 2D analyses under-predict slope safety, due to the resistance at the ends of the failure surface, the so-called 3D-effect, being ignored [19, 29]. Hence, the result of a 3-dimensional assessment of initial slope instability with, for example, the 3D finite element method (FEM), may significantly differ from a 2-dimensional assessment. However, 3D models are often too computationally intensive for general practice. Therefore, various analytical procedures which adjust the results of 2D assessments and require little additional computational cost have been proposed. These methods involve an assumed failure surface geometry in the out-of-plane direction to account for the 3D-effect [1, 19, 29, 30].

For the stability of slopes in spatially variable soils, the method proposed by Vanmarcke [29] has recently been compared against the random finite element method (RFEM) [10, 12, 17, 31]. RFEM accounts for the impact of spatial variability of material properties by combining FEM with random fields for the modelling of spatial variability [4, 7, 10]. Slope failures in RFEM avoid (where possible) local strong zones, thereby reducing the mean factor of safety (FOS) of the slope compared to analyses based only on the point statistics [9]. However, due to a reduced variability of the FOS arising from the spatial averaging of soil properties, the probability of failure computed by RFEM is often lower than the probability of failure from using only the point statistics. Meanwhile, the use of RFEM increases the range of potential failure mechanisms. Compared to RFEM, Vanmarcke’s method overestimates the end resistance and does not account for the effect of weak zones. Varkey et al. [31] highlighted the effect of spatial variability on 3D slope failures and modified Vanmarcke’s method to improve its performance relative to RFEM.

Similar to the differences between 2D and 3D RFEM solutions for initial slope instability, the results of an assessment of the complete failure process would be expected to change if a 3D MPM model were used. 3D RFEM has shown that spatial variability has a significant influence on the likelihood and shape of an initial failure [10, 12, 17, 31], and a large effect of spatial variability on 3D failure processes is therefore to be expected. However, 3D slope failure processes under the influence of spatial variability have not yet been studied in detail, and it is therefore unknown if the analytical procedures to account for the 3D-effect in the initial failure process are valid for the complete failure process. Hence, 3D failure processes, including the effect of spatial variability, are herein studied using the 3D random material point method (RMPM) [20]. In a similar manner to RFEM, RMPM combines random fields with MPM and adjusts the material point properties based on the spatial variability modelled with random fields. Further details on RMPM can be found in Wang et al. [34] and Remmerswaal et al. [21].

This paper provides a first insight into the modelling of 3D slope failure processes and investigates the effect of spatial variability on these processes. A range of failure processes for an idealised problem are presented, together with distributions of both the resisted failure load and failure size. The effects on the failure process of spatial variability in the soil shear strength, as well as a depth trend in the mean shear strength, are studied.

## Three-dimensional slope failure simulation

### Methodology

MPM can be considered a large deformation extension of the finite element method (FEM), where integration points are replaced by material points which are able to move relative to the background mesh, thereby removing mesh tangling restrictions. MPM can therefore be used to model entire dynamic processes, such as those that are evident in retrogressive slope failure. In this paper, MPM is used to model three-dimensional slope failure. While the standard MPM formulation has been shown in the literature to be unstable and inaccurate, enhanced versions have been developed. Here, an optimised implicit MPM scheme with double-mapping and the generalised interpolation material point (GIMP) shape functions (abbreviated as DM-G) has been used [6]. The effect of volumetric locking is reduced using the B-bar approach [39], which has also been successfully applied in FEM. The analyses use the same linear elastic, strain softening plastic, Tresca constitutive model as presented in Remmerswaal et al. [21] to represent the clay soil in the analysed slopes.

The random material point method (RMPM) accounts for the spatial variability of soil properties using random fields [21]. These are numerical predictions of the spatial variability of a soil property, based on the point and spatial statistics of the soil property. The point statistics are the mean ($\mu$) and standard deviation ($\sigma$), which are often combined to give the coefficient of variation ($COV = \sigma /\mu$). The spatial statistic is the scale of fluctuation ($\theta$), which is the distance over which soil property values are significantly correlated in a given direction. In this paper, each random field is generated using local average subdivision (LAS) [4] to give a field of cubic cells, such that each cell corresponds to the initial volume occupied by 1 material point. Hence, the properties of the random field are mapped onto the material points. The same random field is here applied for both the peak and residual undrained shear strengths, i.e. these properties are fully correlated. All other properties are considered to be deterministic. Multiple realisations are used to obtain an overview of the potential failure processes.

For further details of the methodology used in this paper, the reader is referred to references [6, 20, 21, 39].

### Numerical model

**Fig. 1:**
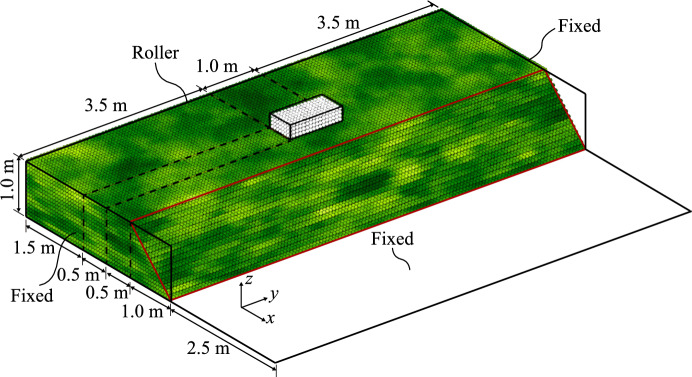
Geometry of the problem domain, indicating the loaded area (with white material points). The material points are coloured according to the undrained shear strength and represent a random field with $\theta _v$ = 0.25 m, $\theta _h$ = 1.25 m and *COV* = 0.25

Figure 1 shows the idealised 1 m high, 45 degree slope, that has been modelled in this investigation. The slope is 8 m wide in the *y*-direction and has a crest dimension (in the *x*-direction) of 2.5 m. Cubic 8-noded finite elements, with an edge dimension of 0.125 m, are used as the computational (background) grid. The elements are initially filled with $2 \times 2 \times 2$ material points evenly distributed within each element. Along the slope face, two material points have been removed from the $x$ $-$ $z$ corner of each element to model the sloping surface. The relatively low number of elements, i.e. 8 elements in the vertical direction, has been used to reduce computational costs to enable Monte Carlo simulations with a reasonable number of realisations. However, care has been taken to ensure that the number of elements is sufficient to reasonably model the spatial variability. Specifically, because random field cell values are assigned at the material point level, there are 4 material points over a vertical distance of $\theta _v$, which satisfies the recommendation in previous RFEM studies of the random field cell size being no greater than $\theta /4$ (e.g. [12]).

The base and ends of the problem domain are fixed in all directions, while the $y$ $-$ $z$ face at the back of the domain prevents movement in the *x*-direction, see Fig. 1. The fixity at the back of the domain does not provide vertical resistance at the boundary, i.e. the estimated strength is conservative. In the *x*-direction, the computational domain extends 2.5 m beyond the toe of the slope, and no fixity is applied to the $y$ $-$ $z$ face at the front of the domain. Material points are removed from the simulation once they exit the domain. Moreover, the $x$ $-$ $z$ faces are free boundaries beyond the toe of the slope. Material points can therefore also leave the domain through the $x$ $-$ $z$ faces beyond the toe. The effect of removing material points is small, since the material loses most of its strength before it reaches these boundaries.

For the Base Case, referred to as Analysis 1, the random fields of peak and undrained shear strength are generated for cubic cells of size 0.0625 m and using a *COV* of the undrained shear strength of 0.25, together with vertical and horizontal scales of fluctuation of $\theta _v$ = 0.25 m and $\theta _h$ = 1.25 m, respectively. Moreover, a mean initial (i.e. peak) cohesion ($\mu _{c_i}$) of 3.6 kPa and a mean residual cohesion ($\mu _{c_r}$) of 0.36 kPa have been adopted, i.e. giving a sensitivity of $S_c = \mu _{c_i}/ \mu _{c_r}$ = 10. The (assumed normal) probability distributions of undrained shear strength have been truncated to prevent negative strengths. This has a negligible effect on the distributions due to the relatively small value of *COV* that is used in the analyses. All other properties are deterministic: the unit weight of the material is 20 $\hbox {kN/m}^3$; the elastic behaviour is governed by a Young’s modulus of 1000 kPa and a Poisson’s ratio of 0.45; and the softening rate is defined by a softening modulus of 2 kPa.

A typical random field realisation from the Base Case is illustrated in Fig. 1. The effects of variations in the horizontal scale of fluctuation, studied after the Base Case, are explained in Sect. 2.3. Each Monte Carlo analysis comprises 300 realisations, and failure is triggered in all realisations, either due only to the slope’s self-weight (i.e. the slope is inherently unstable), or due to the application of a foundation load as described below. Note that although 300 realisations are generally not sufficient for the accurate computation of small failure probabilities, it is sufficient for the qualitative investigation of failure mechanisms carried out in this study. It was also found to be sufficient in previous 3D RFEM studies by Li [16].

The dynamic MPM is solved implicitly using 0.01 s time steps. The occurrence of an inherent instability, i.e. a slope being unstable under its own weight, is first investigated. When the slope is stable, a foundation load is applied until failure is triggered. This ensures that each slope is brought to failure, and thereby minimises the overhead in computing realisations with no failure. Therefore, at the start of the simulation gravity loading is applied in an elastic implicit quasi-static MPM step to generate 99% of the initial (i.e. in situ) stresses, with movement of the material points and plasticity being prevented. The remaining 1% is applied at the start of the simulation, whereupon movement and plasticity of the material points are allowed, which may trigger an inherent instability. For cases in which the slope is stable under its own weight, an increasing load is applied to the slope crest through the foundation, by increasing the weight of the material points representing the foundation. This load is analogous to the build-up of material on top of a 1 m by 0.5 m rectangular area, located 0.5 m from the slope crest. This foundation has been modelled as a linear-elastic material with the same elastic properties as the slope, i.e. a Young’s modulus of 1000 kPa and a Poisson’s ratio of 0.45.

**Fig. 2:**
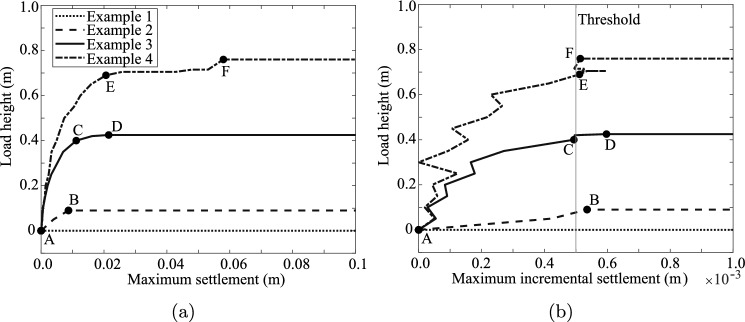
The loading scheme in four example realisations. The load height as a function of **a** maximum settlement, and **b** maximum incremental settlement. Note that the results are plotted every 10 time steps

In each time step, an additional load equivalent to 0.005 m depth of soil is applied, unless the incremental settlement in the previous time step exceeded a threshold of 0.0005 m (in which case, no increment of load is applied). The incremental settlement threshold is used to prevent overloading of the slope beyond its actual failure capacity. The incremental settlement of material points during a time step is equivalent to the velocity of the material points; hence, the load is only increased when the velocity is low. An incremental settlement threshold is used instead of a total settlement threshold, since the total settlement to reach failure can vary significantly between realisations (see Fig. 2a).

The loading scheme is further explained by the examples shown in Fig. 2. The slope in Example 1 is inherently unstable, and the maximum incremental settlement exceeds the threshold without the application of a foundation load. During the analysis, the displacement increased far beyond the 0.1 m limit of Fig. 2a. In Example 2, the load is increased from point A until the failure capacity is reached at point B. From point B the maximum incremental settlement continuously exceeds the threshold. In Example 3, the load is increased from points A to C. Loading is paused at C because the maximum incremental settlement in the previous step exceeded the threshold. In other words, the velocity of the slope was too high. However, the failure capacity has not yet been reached at point C, i.e. the incremental settlement decreases with time under the same load and, without a further load increase, the slope becomes stable again before large deformations can occur. Between C and D, the foundation load is increased whenever the incremental settlement in the previous step is below the threshold. So, whenever the material points slow down enough, additional load is applied until the failure capacity is reached (point D in Example 3), after which the material points continuously accelerate up to large deformations.

A similar behaviour is observed in Example 4, where the load is increased continuously until point E. At this load, the incremental settlement increased significantly beyond the threshold. However, the incremental settlement decreased again in later time steps, i.e. the failure capacity was not reached at point E. The load is then increased whenever the material points slow down to below the threshold until reaching the failure capacity at point F.

When the sliding mass slows down towards the end of the failure process, the incremental settlement decreases again. Figure 2b is terminated once the failure has been fully developed, and so the eventual decrease in incremental settlement is not shown. To prevent further loading after the failure has occurred, loading is no longer increased once the maximum total settlement exceeds 0.1 m. However, secondary failures can still occur after the initial failure without further loading.

**Table 1:** Model details

Geometry	Discretisation	Material properties	
*H* = 1 m	$\Delta t$ = 0.01 s	$\gamma$ = 20 $\hbox {kN/m}^3$	$c_i$ = N($\mu _{ci}$, *COV*)
*W* = 2.5 m	$t_{max}$ = 15 s	*E* = 1000 kPa	$c_r$ = $c_i$/$S_c$
*L* = 8.0 m	$\Delta x$ = 0.125 m	$\nu$ = 0.45	$S_c$ = 10
Slope 1:1	$\Delta y$ = 0.125 m	$H_S$ = −2 kPa	$\theta _v$ = 0.25 m
	$\Delta z$ = 0.125 m		0.25 m $< \theta _h <$ 10.0 m

**Table 2:** Summary of analyses

Analysis	Comments	${\mu }_{{c_i}}$ (kPa)	*COV* (–)	${\theta }_{{h}}$ (m)	*k* (kPa/m)
1	Base Case	3.6	0.25	1.25	0
2A	Influence of horizontal scale of fluctuation	3.6	0.25	0.25	0
2B		3.6	0.25	2.5	0
2C		3.6	0.25	5.0	0
2D		3.6	0.25	10.0	0

3A	Influence of depth trend in mean shear strength	3.6	0.25	1.25	3.0
3B		3.6	0.25	1.25	6.0

### Overview of analyses

The properties of the Base Case (referred to in the previous section) are summarised in Table 1 and the first row of Table 2. The other rows in Table 2 present variations with respect to the Base Case. Hence, Analysis Sets 2 and 3 investigate the influence of the horizontal scale of fluctuation and the linear depth trend (*k*) in the mean undrained shear strength, respectively. The failure processes computed for the Base Case are compared against the results obtained for these other analysis sets.

Note that while the initial mean shear strength was chosen to give a relatively high probability of inherent slope failure for the slope height analysed, the *COV* is within the expected range found in the literature [11] and the value of $S_c$ is within the expected range for medium sensitive clays (e.g. [15]). Moreover, the value of $\theta _v$ is typical for clayey soils [38], while the investigated range of $\theta _h$ covers typical ratios that may be encountered for $\theta _h/\theta _v$ [38], i.e. from isotropic spatial variability to $\theta _h$ exceeding the problem geometry.

### Quantifying the failure volume

Hicks et al. [8, 13] computed estimates of failure volume to quantify the failure consequence in their 3D RFEM analyses of slope reliability. These were based on calibrating a threshold displacement beyond which failure was deemed to have occurred. Meanwhile, in the post-processing of 2D RFEM analyses of slope reliability using subset simulation, van den Eijnden and Hicks [28] separated the stable material from the unstable material using the K-means clustering method (KMCM) to estimate slide volumes.

The KMCM approach has here been modified for RMPM. In RFEM, two clusters are enough to separate the sliding mass from the stable mass (i.e. the sliding mass is one cluster and the stable mass is the other cluster). However, in RMPM, the large differences in deformations between the initial and secondary failures cause the clustering with two clusters to be unreliable, as secondary failures may be erroneously clustered with the stable material instead of with the sliding mass.

**Fig. 3:**
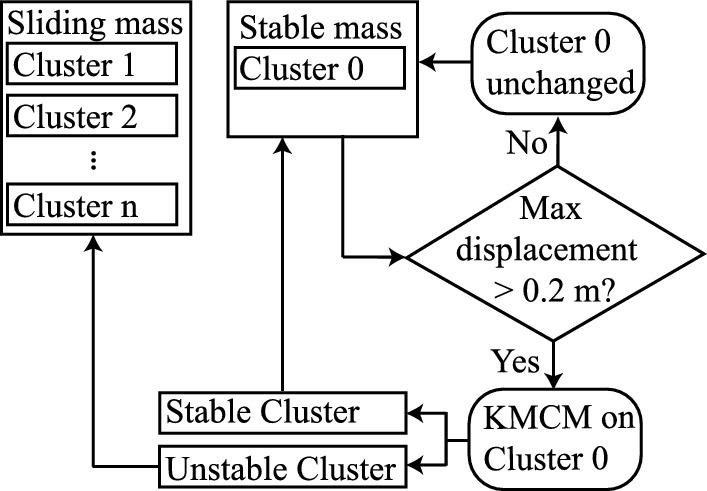
Flow chart for the sub-clustering KMCM algorithm

KMCM is, therefore, not used during the post-processing at the end of the simulation, but instead during the simulation itself, whenever an initial or subsequent failure occurs. Figure 3 shows a flowchart for this algorithm. At the start of the simulation one stable cluster exists (Cluster 0). When a failure occurs, KMCM separates the sliding mass from the stable mass (i.e. the cluster containing the stable mass is split into two new clusters: a new sliding mass and a new stable mass). When KMCM is used again later in the analysis, i.e. following another secondary failure, only the remaining stable mass is split into new clusters. So, during the analysis the size of the stable cluster reduces as more unstable clusters are detected. Meanwhile, the number of unstable clusters gradually increases. The unstable clusters, i.e. the clusters with sliding masses, remain unchanged once formed.

To detect if a failure has occurred, i.e. to detect when KMCM should be used to register a new sliding mass, the maximum Euclidean displacement of the remaining stable material points ($u_{stable,max}$) is computed. Failure is deemed to have occurred whenever $u_{stable,max}$ exceeds a (user-defined) threshold, here set to be 0.2 m (i.e. one fifth of the slope height), see Fig. 3. The threshold $u_{stable,max}>$ 0.2 m ensures an accurate division of each failure for this problem, and has been established based on the visual inspection of several realisations.

By the end of the simulation, the material may have been divided into any number of clusters. Each cluster contains either the remaining stable mass, the sliding mass of the initial failure, or the sliding mass of a subsequent failure. The clusters are used to estimate (1) the stable volume, (2) the total failed volume, (3) the retrogression distance (in the *x*-direction), and (4) the damaged crest width (in the *y*-direction). The retrogression distance is defined as the largest distance from the crest until a stable material point, whereas the damaged crest width is defined as the total width (in the *y*-direction) of all failed crest material points. Although this modified KMCM procedure also computes the failed volumes of individual slides, these results are not discussed here.

## Analysis 1: Base Case

### Failure processes of inherently unstable slopes

**Fig. 4:**
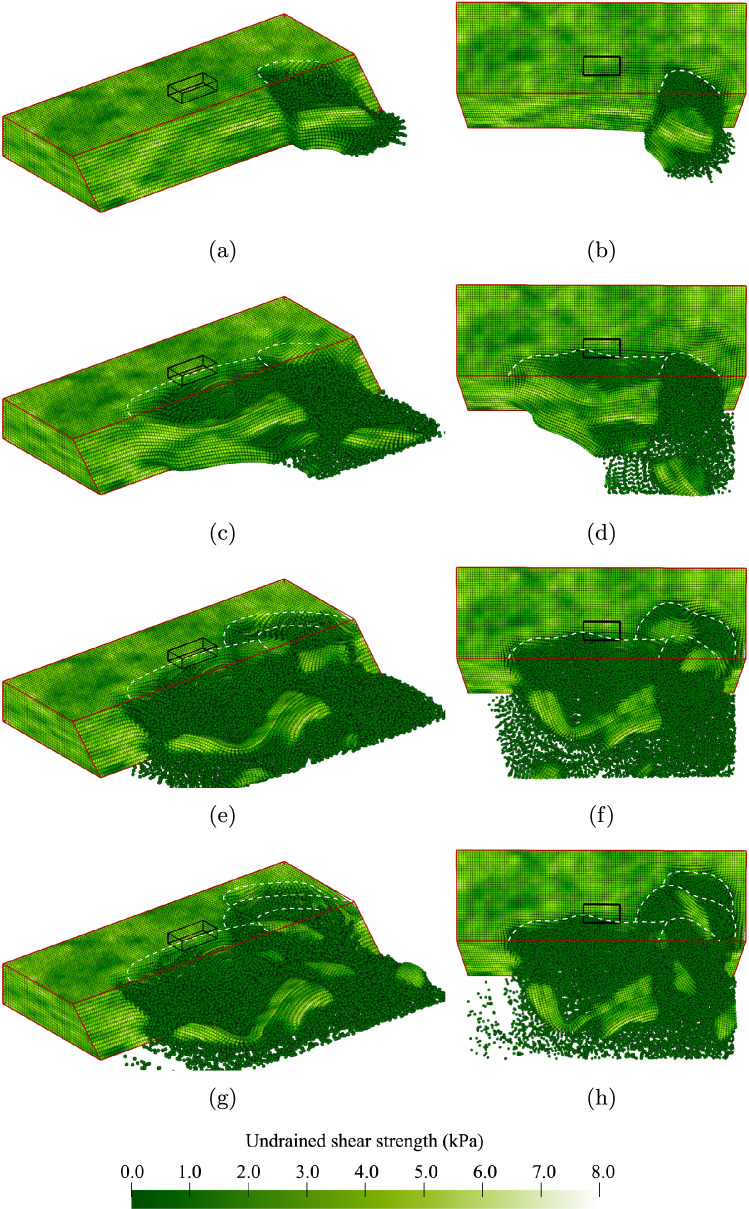
An inherently unstable slope failure: **a** and **b** small initial failure; **c** and **d** sidewards spreading of the failure; **e** and **f** large retrogression backwards; **g** and **h** end of the simulation

Some of the slopes are inherently unstable due to a weak zone within the slope (10.4% of the slopes in Analysis 1). The probability of initial failure is high on purpose to study the behaviour after inherent instabilities. An example of this behaviour is illustrated by the contours of undrained shear strength shown in Fig. 4. Note that because large deformations are accompanied by strain softening (i.e. a reduction in undrained shear strength), the developing failure process is indicated by the spreading of darker zones during the analysis. The outline of the undeformed slope is highlighted in red and the centrally located surface load at the slope crest, which is not applied in this simulation, is highlighted in black. Ridge lines are indicatively drawn as broken white lines to highlight the location of each failure surface with respect to the original slope crest. For each (selected) time step, 3-dimensional and top views are presented.

Figure 4a, b shows the same small initial failure of approximately 2 m in width near the far end of the slope, i.e. the centre line of the failure is located around 6.5 m from the left-hand boundary. The initial failure triggers a large instability along the remainder of the slope, as shown in Fig. 4c, d. This second instability is constrained by the presence of a strong zone at the toe of the slope between 1.0 m and 2.0 m from the left-hand boundary. The initial failure also triggers a retrogressive mechanism towards the back of the slope. First, a third slide of similar size to the initial failure occurs, as shown in Fig. 4e, f. Then, a smaller fourth slide can be observed in Fig. 4g, h. The fixed end point of the simulation after 15 s is reached in Fig. 4g, h. By this end point, the deformations have slowed down and it is therefore unlikely that additional failures would occur if the simulation were continued beyond 15 s.

Figure 4 highlights the importance of modelling the failure process in 3D, in that the failure process is clearly 3-dimensional. Moreover, the importance of modelling the entire failure process becomes clear. Based on the size of the initial failure, a relatively small consequence could be attributed to the slope failure; however, the retrogressive and 3D nature of the full failure indicates a more severe consequence in this instance.

### Failure processes triggered by a foundation load

**Fig. 5:**
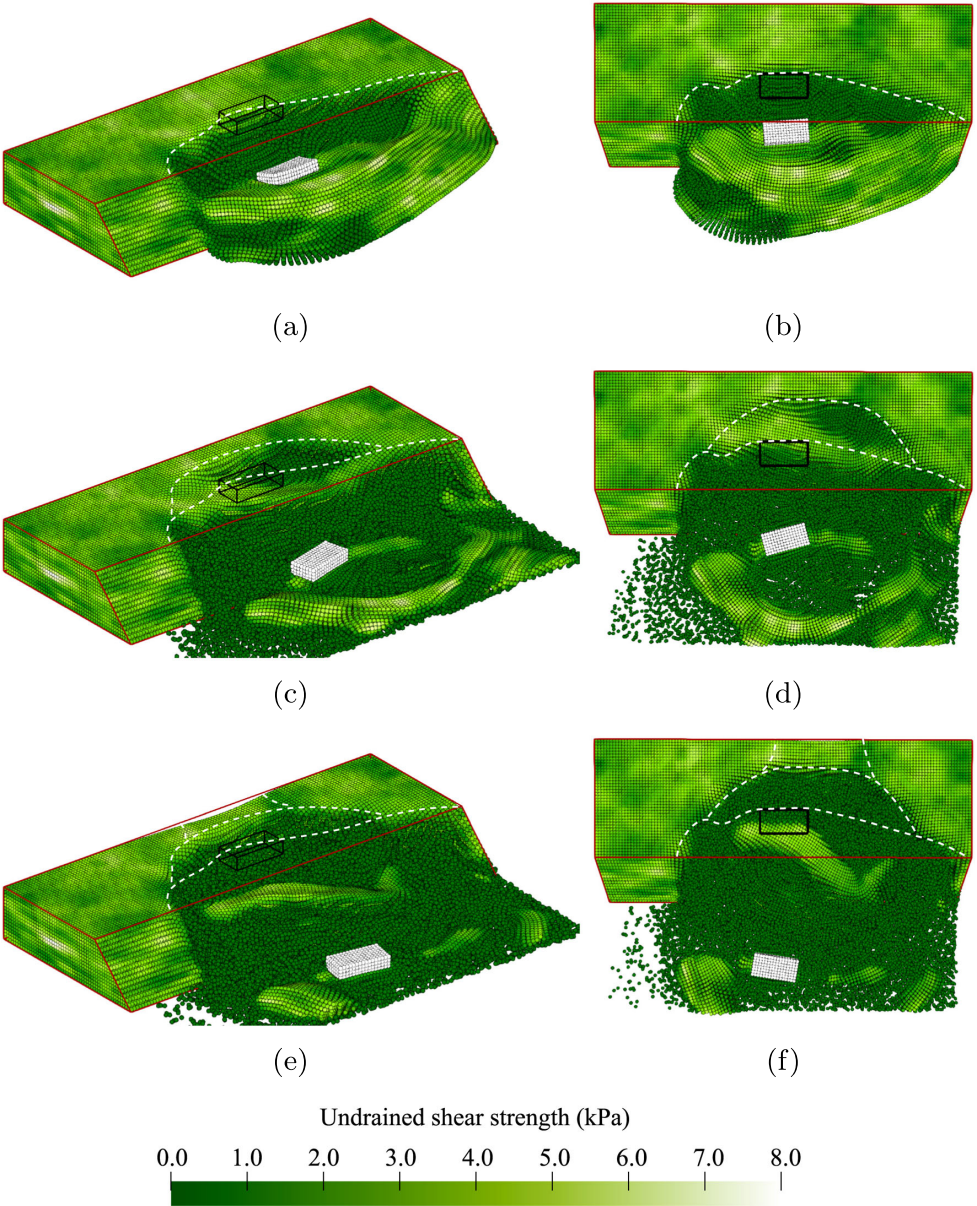
A slope failure due to the applied foundation load: **a** and **b** large slightly asymmetric initial failure; **c** and **d** backwards retrogressive failure; **e** and **f** retrogressive failure fully developed and a third deformation zone initiates at the back of the slope before the end of the simulation

Most of the slope simulations are stable under their own weight (89.6% of the slopes in Analysis 1). The surface load is then applied to trigger slope failure, with an example illustrated in Fig. 5. The fixed location of the surface load at the centre of the slope dictates the location of the failure initiation along the slope, i.e. failure initiates near the load. However, the size of failure can vary. Figure 5a, b shows that, in this example, a 6-m-wide initial failure occurred, which has a bowl-shape similar to the inherently unstable failures shown in Fig. 4.

The initial slide is followed by a slightly smaller secondary slide, see Fig. 5c, d. The first slide initiated below the surface load and triggered an asymmetric failure (i.e. a larger failure to the right of the load than to the left). The second slide initiates 1 m to the right of the location of the load, i.e. it follows the asymmetric geometry of the first failure. By the end of the simulation (Fig. 5e, f), the second failure has spread to become approximately the same width as the initial failure, and a small third failure has initiated in the remainder of the slope towards the fixed boundary, i.e. the slope height at the fixed boundary has fallen, as evident in Fig. 5e.

**Fig. 6:**
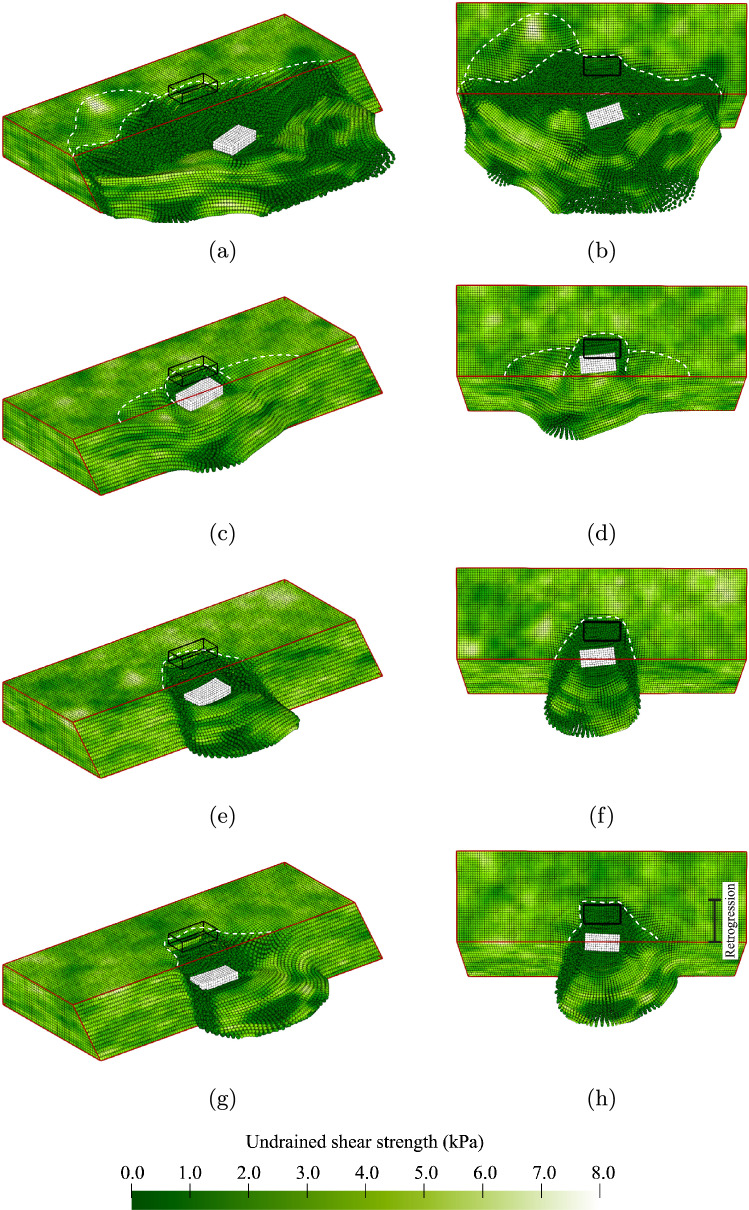
Various possible initial failures due to a foundation load: **a** and **b** failure width extends over whole domain; **c** and **d** failure width roughly half the slope width; **e** and **f** failure width roughly equal to surface load width; **g** and **h** asymmetric failure surfaces bounded by a strong zone on one side of the surface load

Although the use of a surface load as the trigger reduces the variation in the location of failure initiation, more variation in the subsequent failure process can be observed. Moreover, a large variation in the failure width is possible even when a surface load is applied, as illustrated in Fig. 6. The surface load can trigger failure along the entire width of the slope when the slope is barely stable under its own weight and the strength of the material is approximately constant along the entire slope (Fig. 6a, b). However, stronger zones usually occur along the slope width. Local deformations around the load, similar to a bearing capacity failure, may then occur, and a further increase in load triggers slope failure, see Fig. 6c, d. The strong zones may even reduce the failure surface extent to the width of the surface load (Fig. 6e, f). When strong zones are only present on one side of the surface load, an asymmetric failure, as shown in Fig. 6g, h, can be triggered.

**Fig. 7:**
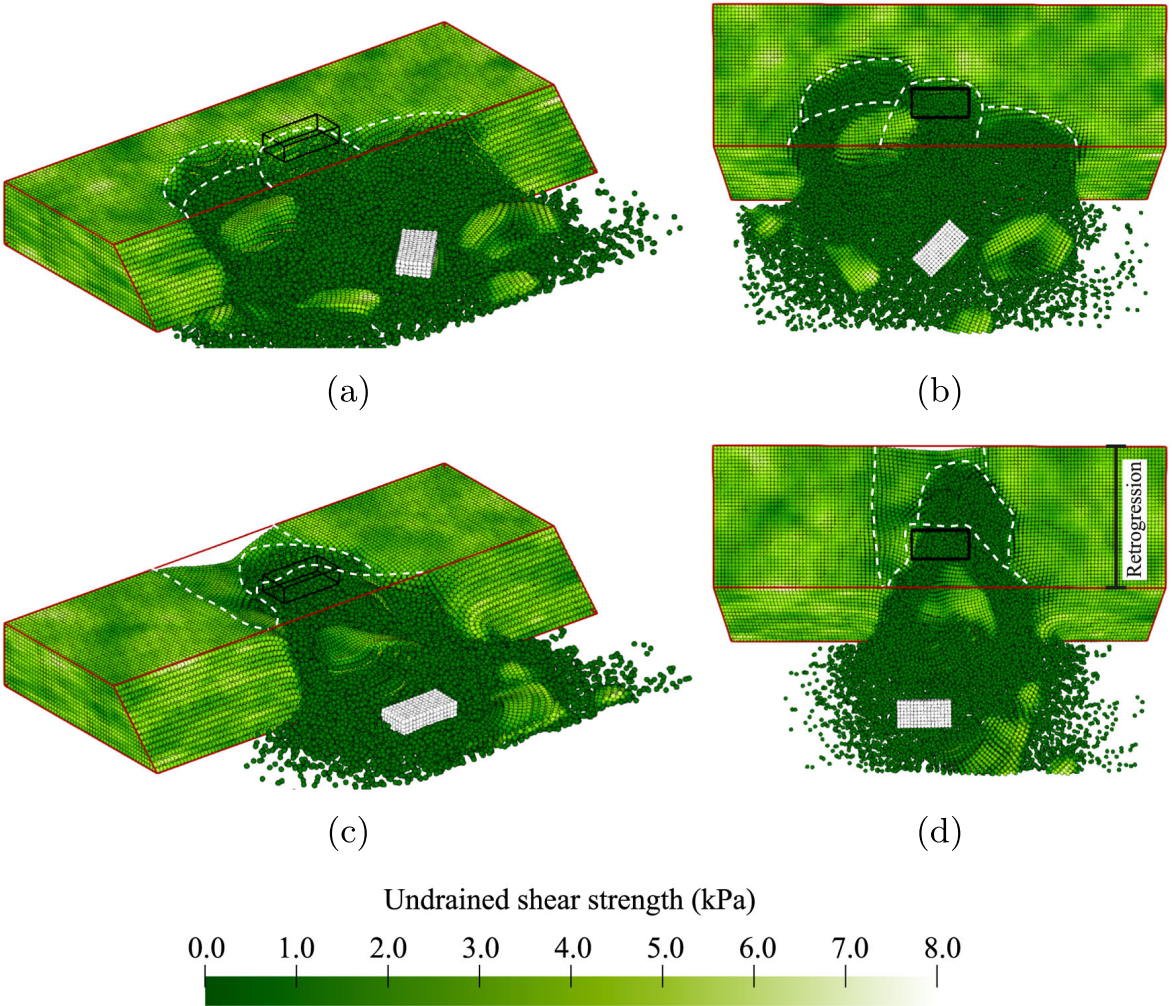
Final slide configurations in 2 realisations: **a** and **b** failure process predominantly parallel to the slope after the initial failure shown in Fig. 6c, d; **c** and **d** mostly backwards failure process after the initial failure shown in Fig. 6g, h

Although Fig. 5 shows retrogressive behaviour mainly towards the back of the domain, retrogressive behaviour can move in multiple directions in 3D. For example, Fig. 7 shows the final configuration after two different failure processes. During the initial failure shown in Fig. 6c, d, the sides of the initial failure are pulled with the moving material, thereby widening the failure, see Fig. 7a, b. After the sidewards extension, a smaller block at the back wall of the failed area becomes unstable, also shown in Fig. 7a, b. In this specific case, the momentum of the block is not large enough for it to flow out of the domain, and it instead comes to rest in the failure zone.

After an initial failure with a smaller width, such as the asymmetric failure shown in Fig. 6g, a third and less likely failure process may occur: that is, retrogressive failures may form a small tunnel away from the slope face from the gap created by the initial failure. The tunnel shown in Fig. 7c, d tends to get smaller with each subsequent failure. Not all the material can flow out of the tunnel, as the material still has some undrained shear strength and rests on a horizontal fixed boundary. This remaining material has a stabilising effect, which causes the tunnel to narrow. One may expect instabilities at the sides of the tunnel when (1) the material is capable of flowing out, or (2) when the sides of the tunnel have a weak zone. The chance of encountering a weak zone at the sides of the tunnel would increase were the tunnel able to progress further beyond the back boundary included in this model.

### Failure initiation and process

**Fig. 8:**
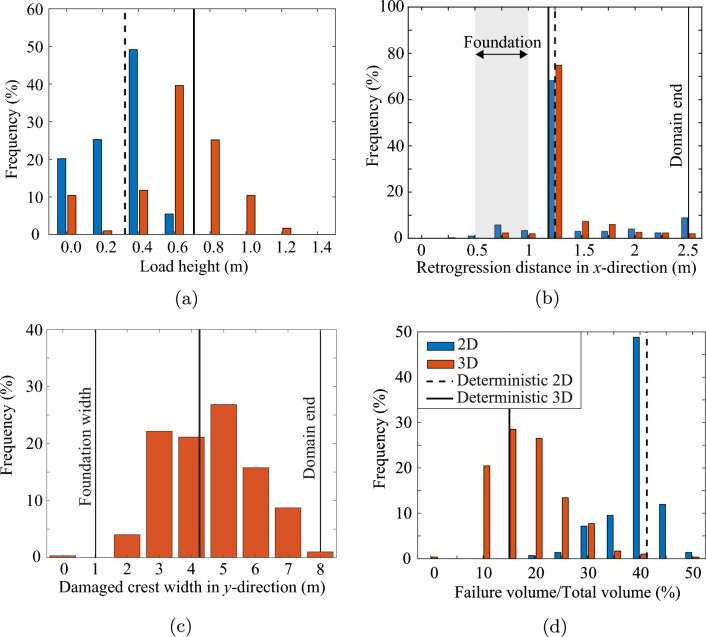
**a** Distribution of the ultimate foundation load, represented as the height of soil built up on the foundation, for Analysis 1; **b** distribution of the retrogression distance, the largest distance from the initial slope crest to the failure surface (measured along the crest) for Analysis 1; **c** damaged crest width for Analysis 1; and **d** relative failure volume for Analysis 1. Comparative 2D simulations using the same statistics are also presented

Figure 8a shows the distribution of ultimate foundation loads for all the realisations in the Base Case. The ultimate load heights are placed into bins of 0.2 m intervals, where the label in the figure indicates the average value of a bin. The first bin contains load heights from 0.0 to 0.1 m, but is centred on 0.0 as it mainly contains the realisations with zero load height, i.e. 10.4% of the 3D slopes are inherently unstable. A wide spread around the 2D and 3D deterministic results (i.e. based on the mean property value) is observed, where some slopes are unable to resist their own self-weight (foundation load of 0 m), while others can resist a foundation load equivalent to more than the slope height.

For comparison, 2D simulations were performed for the cross section through the middle of the slope. The 2D simulations use the same random fields, i.e. from each 3D random field the centre cross section was selected and used to perform the 2D analysis. In 2D, more slopes are inherently unstable (20.1%) compared to 3D and the resisted foundation load on inherently stable 2D slopes is significantly lower on average. Hence, the fact that a failure in 3D can potentially occur at more weak locations is more than compensated for by the stabilising effect of the sides of a failure surface in 3D for the slope geometry and material properties considered in this investigation.

Figure 8b shows the distribution of the final retrogressive distance, measured from the slope crest in the *x*-direction, as well as the location of the foundation in the *x*-direction. It shows a large peak at 1.25 m, i.e. the most likely size of the initial failure mechanism, indicating that retrogressive behaviour in the *x*-direction, i.e. away from the crest, is unlikely. The local peak at around 0.8 m is caused by inherently unstable slopes, and is especially visible in the 2D simulations, where more slopes are inherently unstable (see Fig. 8a). Even though retrogressive behaviour is unlikely, when it does occur it can significantly increase the damage to the slope, potentially even reaching the end of the domain at 2.5 m. When retrogressive behaviour occurs in 2D, it is more severe compared to 3D, indicating a stabilising 3-dimensional effect on the retrogressive behaviour. The stabilising effect also compensates for more potential failure paths in 3D. Even though a 2D simulation may miss 3-dimensional processes, it is likely to be a conservative estimate of the retrogression distance.

Figure 8c shows that much more variation is present in the failure process in the *y*-direction (parallel to the slope). A deterministic analysis computes a failure width of around 4.2 m, i.e. approximately 4 times both the slope height and width of the foundation load. Moreover, the deterministic analysis often under predicts the failure width. Figure 8d shows that a 3D deterministic analysis can underestimate the failure volume by a factor of 2 to 3. The failure volume of the 2D simulations has also been computed, where failure is assumed to occur along the entire width of the 3D domain. This is a conservative assumption, given that failures would (in reality) have a limited width in the third dimension. The 2D analysis significantly over predicts the failure volume under this assumption, with a higher peak at around the deterministic solution. For improved visibility, the figure is limited to a relative failure volume of 50% (of the domain volume), although 20% of the 2D simulations have a relative failure volume above 50%, with a fairly uniform distribution from 50% to 100% relative failure volume. This again indicates that retrogressive behaviour is more extensive in 2D analyses.

## Analysis Set 2: influence of horizontal scale of fluctuation

**Fig. 9:**
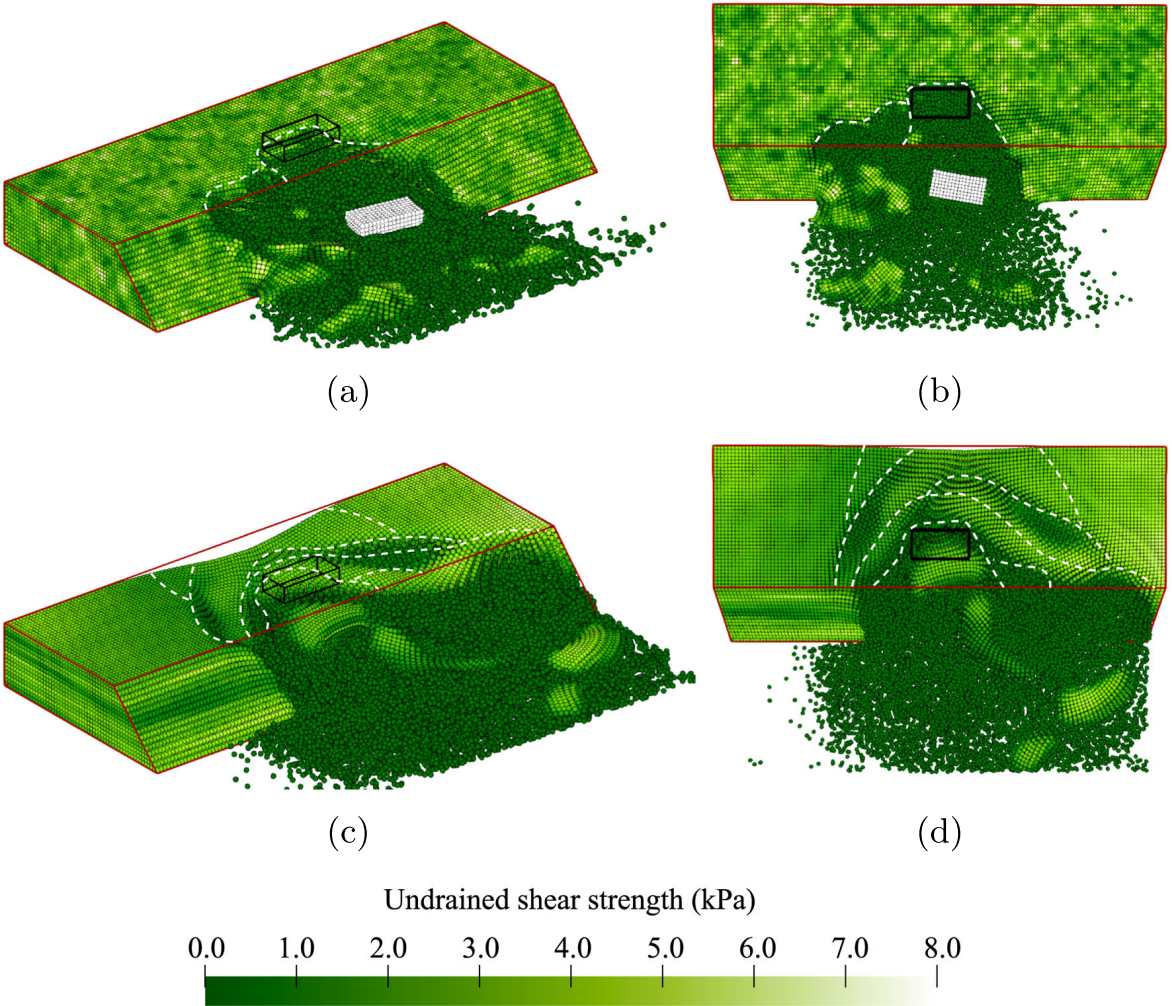
Example failures showing influence of degree of anisotropy of the soil heterogeneity: **a** and **b** $\xi$ = 1, i.e. no layering; **c** and **d** $\xi$ = 40, i.e. layers more extensive than the length of the slope

Analysis Set 2 describes the failure process for a full range of values of the horizontal scale of fluctuation. Figure 9a, b shows a realisation with a degree of anisotropy of the heterogeneity ($\xi = \theta _h/\theta _v$) equal to 1 (with $\theta _h$ = $\theta _v$ = 0.25 m), i.e. a realisation from Analysis 2A. The failure initiation is similar to Fig. 7c, d, where the width of failure at the load is approximately equal to the 1 m width of the loaded area and expands to 2 to 3 m wide at the base of the slope. These failures with a small width occur with a high frequency for low degrees of anisotropy (coupled with a relatively small value of $\theta _h$), as the outcomes approach the deterministic solution. After the initial failure, and due to the high variation within the soil, the material breaks into smaller blocks compared to realisations in the Base Case. This results in a more chaotic remaining profile, see Fig. 9a, b, while smoother profiles are observed in the Base Case.

A realisation with a degree of anisotropy of 40 (from Analysis 2D) can trigger failure above the base of the slope. Due to a large weak layer, this kind of failure often triggers the almost complete collapse of the slope once failure occurs (as shown in Fig. 9c, d). Figure 9c, d shows that the initial failure causes settlement in a large area surrounding the foundation, which is quickly followed by large retrogressive failures where both the sides and back of the failed area are pulled in with the initial failure. Large intact blocks remain in the failure zone as failure blocks slide down. These blocks are usually larger in the *y*-direction than in the *x*-direction, as the failure surface perpendicular to the slope still tends to be circular in the *x*-direction, limiting the failure size.

### Failure initiation and process

**Fig. 10:**
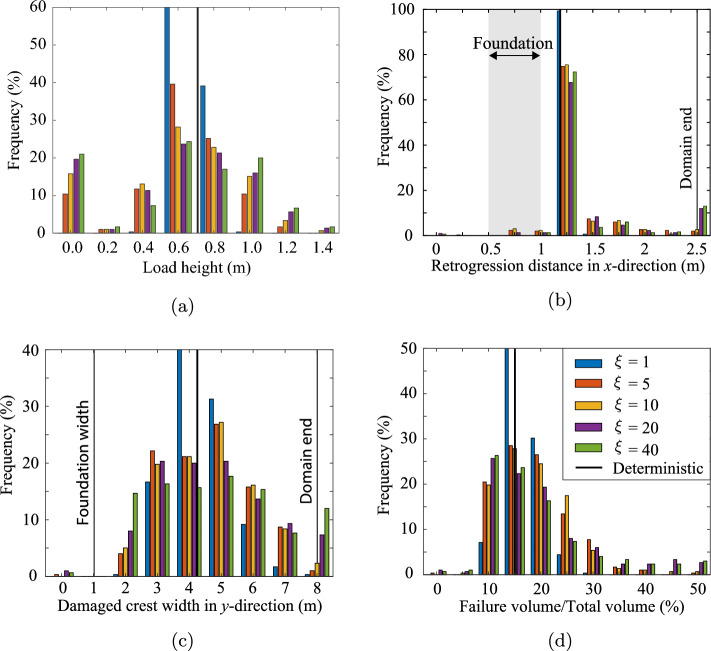
Effect of degree of anisotropy ($\xi$) of the soil heterogeneity on **a** the ultimate foundation load, **b** the retrogression distance, **c** the damaged crest width, and **d** the relative failure volume

Figure 10a shows the effect of the horizontal scale of fluctuation on the distribution of the ultimate limit load. For a degree of anisotropy of $\xi$ = 1, i.e. no layering of the soil heterogeneity, there is limited variation around the deterministic solution based on the mean strength properties. This is because of the significant averaging of material properties along the failure surface, as can be expected for this degree of anisotropy and adopted value of $\theta _v$. Moreover, inherent failures cannot be triggered, because the weak zones in the material are too small to promote development of failure mechanisms that avoid the stronger zones. At the other end of the spectrum, a degree of anisotropy of $\xi =$ 40 shows a large variability in the failure load. In some cases, strong zones are present at the base of the slope where the loads due to gravity loading are at their highest, thereby providing the ability to resist a larger failure load. A strong zone can even force failure initiation through a weak layer above the base of the slope. Conversely, a weak zone along the base often triggers an inherent instability. Intermediate degrees of anisotropy confirm that the influence of strong and weak zones increases with an increase in the degree of anisotropy. In other words, larger strong zones can more often lead to an increase in the resisted load than smaller strong zones, while larger weak zones are more likely to trigger inherent instabilities than smaller weak zones.

The variation in the retrogression distance increases with an increase in the degree of anisotropy, as shown in Fig. 10b. In many cases, no retrogressive behaviour in the direction away from the slope is observed and a large peak is present between 1 and 1.5 m retrogression distance. A high degree of anisotropy causes more inherent instabilities compared to a low degree of anisotropy. Moreover, retrogressive failures tend to be more likely for higher degrees of anisotropy, and retrogressive behaviour combined with significant layering is likely to cause the complete slope to collapse.

Figure 10c shows the variation in the damaged crest width for the various degrees of anisotropy. Figure 10c indicates that strong zones in a layered material can have a limiting effect on the width of the initial failure surface, while retrogressive behaviour through weak zones can trigger full collapse of the slope. The responses for a small amount of layering ($\xi$ = 5) and a large amount of layering ($\xi$ = 40) are similar, while no layering clearly shows less variation. In the case of no layering ($\xi$ = 1), failures tend to have a size that is closer to the deterministic solution.

Figure 10d shows the influence of $\xi$ on failure volume. The figure highlights that, for larger values of $\xi$, there is an increased likelihood of retrogressive failure leading to very large failure volumes, as seen from the tails of the histograms. Note that although the figure indicates relatively few large failures of this type, around 6% and 10% of the simulations for, respectively, $\xi =20$ and $\xi =40$, have relative failure volumes of 50–100%. However, these results are not included in the figure for reasons of clarity. For $\xi$ smaller than or equal to 10, fewer than 1% of the simulations have relative failure volumes exceeding 50%.

## Analysis Set 3: depth trend in the mean shear strength

**Fig. 11:**
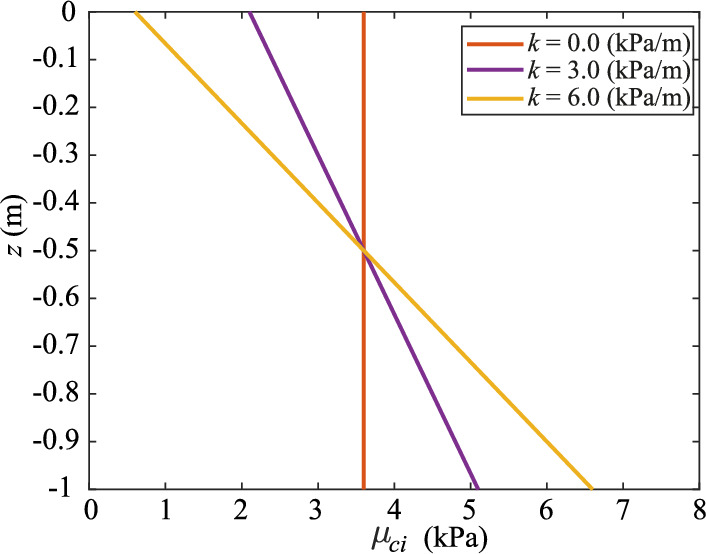
Mean undrained shear strength at a given depth as a function of the depth trend *k*

In Analysis Set 3, a linear depth trend (*k*) in the mean undrained shear strength is introduced, i.e. in this case the mean undrained shear strength increases linearly with depth. The results of the Base Case without a depth trend, i.e. *k* = 0 kPa/m, are compared against *k* = 3.0 kPa/m (Analysis 3A) and *k* = 6.0 kPa/m (Analysis 3B). The depth average of the undrained shear strength is the same in all analyses as shown in Fig. 11.

**Fig. 12:**
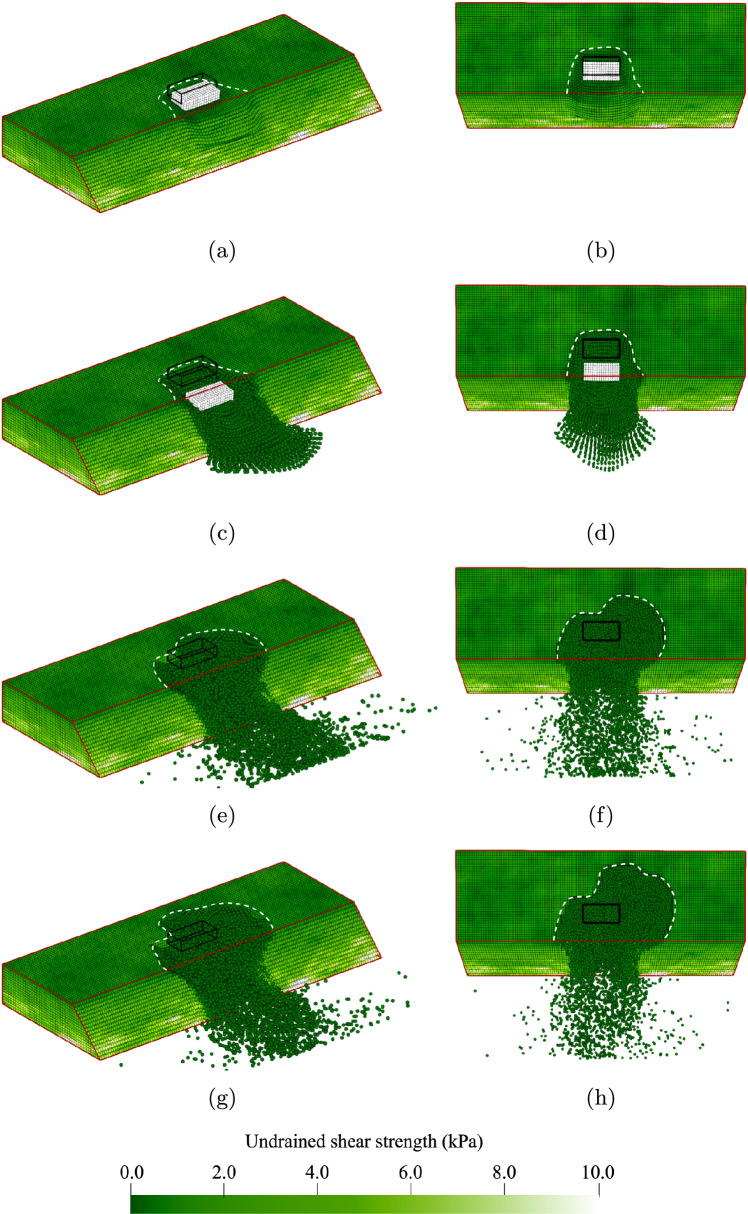
Example of a failure with a depth trend *k* = 6.0 kPa/m in the mean undrained shear strength: **a** and **b** initial failure after loading through a weak zone halfway up the slope; **c** and **d** small fully developed initial failure, where the base of the slope remains stable; **e** and **f** flow-like retrogressive behaviour of the weak material at the top of the slope; **g** and **h** end of the simulation after more flow-like retrogressive behaviour

One example of a failure process with a large depth trend (*k* = 6.0 kPa/m) is shown in Fig. 12. Figure 12a, b shows that the initial failure due to the foundation load occurs through a weaker zone approximately halfway up the slope. Due to the fact that the failure is shallow, the size of the failure is relatively small (Fig. 12c, d). Compared to a material without a depth trend, retrogressive behaviour does not occur along clearly defined slip planes. Instead, as shown in Fig. 12e–h, the material appears to flow steadily along a gentle slope into the failure zone. Instabilities at the sides of the initial failure, which tend to occur frequently in materials without a depth trend, are much less frequent in a material with a depth trend, i.e. retrogressive failure occurs more in the direction away from the slope and less along the slope.

**Fig. 13:**
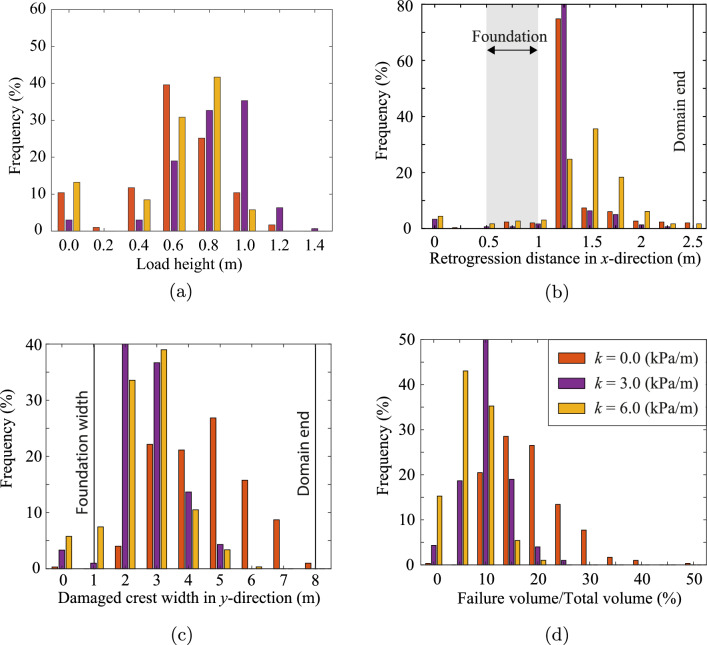
**a** Load height, **b** retrogression distance, **c** damaged crest width, and **d** relative failure volume for Analysis Set 3 with and without a depth trend in the mean undrained shear strength

Figure 13a shows the effect of a depth trend in the mean undrained shear strength on the range of resisted loads. As the strength at the base of the slope, where the gravity loads are highest, increases with an increase in *k* to 3.0 kPa/m, the resistance of the slope against inherent instabilities and foundation load increases. This is because deep failures are critical, so that the safety against failure increases when the resistance increases with depth near the slope toe. However, as *k* increases further, the influence of the slope height reduces, until, at *k* = 6.0 kPa/m, the tendency to fail becomes independent of slope height for a homogeneous soil. This means that, for a soil that is spatially variable about the mean strength, there is an increased likelihood of shallow failures, and this increased likelihood of shallow failures counteracts the increased resistance at the base of the slope. The overall resistance of the slope (including heterogeneity) for a high value of *k* is, for this specific case, lower than for a smaller value of *k*. Hence, a limited depth trend can raise the resistance, since the higher strengths at greater depths can resist the critical deep failures, while a larger depth trend increases the possibility of failures along planes at different depths, thereby reducing the overall resistance.

A similar effect can be observed in the extent of the failure process, as shown in Fig. 13b: a small depth trend reduces retrogressive behaviour, whereas a large depth trend increases retrogressive behaviour due to the increased possibility of failure at multiple depths.

**Fig. 14:**
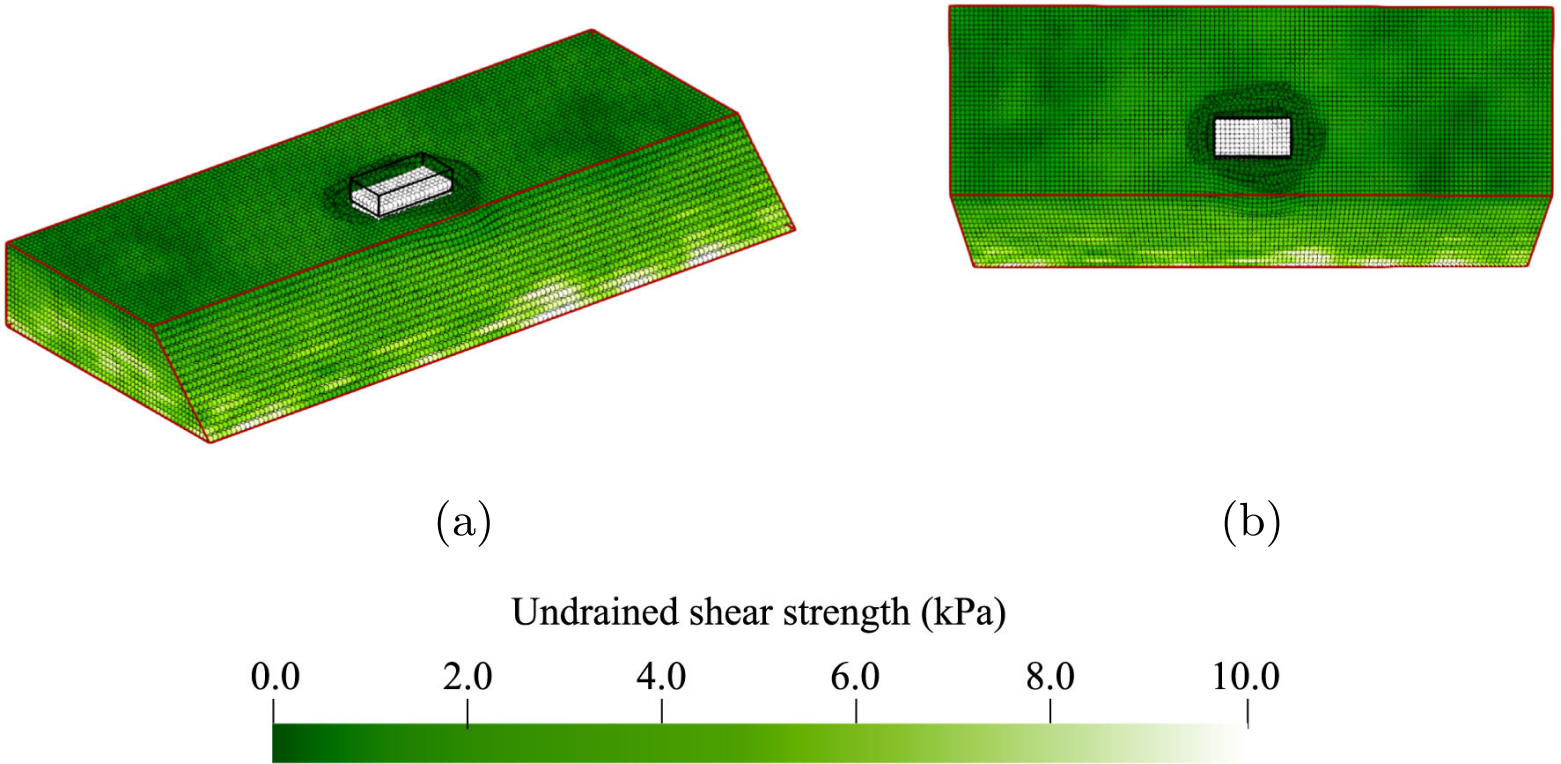
Final geometry of a foundation failure on a slope with a depth trend *k* = 6.0 kPa/m in the mean undrained shear strength

Figure 13c, d confirms that slides with a depth trend are smaller on average, as the slides can occur through layers above the base and tend to spread less in the direction along the slope. Although, in the analyses discussed previously, foundation failure only occurred at the same time as the slope failure, with a significant depth trend a foundation failure can sometimes occur even though the slope remains stable (see Fig. 14). This can occur due to significantly weaker material near the crest of the slope. The crest can remain almost intact with this failure mechanism, with the failure volume then tending to be small in comparison with volumes involved in a slope failure mechanism.

## Discussion

This study has considered an idealised slope stability problem to explore possible failure processes, and to highlight the potential of 3D RMPM as an effective analysis tool. The slope geometry is similar to previous slopes analysed using RFEM, so that comparisons between studies are possible. In addition, slides in sensitive clay have been known to initiate in relatively small (e.g. lake-side) slopes. The study has provided valuable insight into how 3D failure mechanisms evolve. An obvious next step is to consider real case histories and slope geometries, as well as more realistic constitutive models of soil behaviour.

The simulations were performed in parallel on the grid computing system Spider at SURFsara, and the model size was chosen so that each realisation could be analysed on a single computer processor of this system. Each realisation took 12–24 h of computer run time. Hence, when using 150 computer processors, simulations involving 300 realisations could be completed within a couple of days.

It was found that 300 realisations were sufficient for quantifying the trends in failure processes, as is evidenced by the consistency in the histogram results. However, for the accurate computation of failure probability (especially at the weak tails of the distributions) more realisations would be needed, requiring code optimisation or special strategies such as subset simulation. In addition, to model real slopes, more complex geometries must be created, for which an increase in the required number of elements and material points is expected. The current 3D RMPM code uses a standard implicit time integration scheme, although this can be optimized to reduce the memory and computation time footprint.

Note that the use of 3D RMPM in engineering practice is, for now, not suggested by the authors (i.e. using current computational machines). However, the methodology, especially after numerical optimisation, is useful for better understanding failure processes within a scientific framework, where grid computing is often available. It can, for example, also be used to derive analytical/numerical frameworks for including 3D-effects within 2D (R)MPM simulations.

## Conclusion

3D RMPM has been shown to be capable of producing an overview of many potential failure processes, and quantifying the failure consequences of these processes. It can provide insight into the effect of spatially varying shear strength properties on the failure onset and consequence. The so-called 3D-effect increases the safety against the onset of failure (as is also well recognised from FEM analyses) and reduces the likelihood and size of secondary failures compared to 2D RMPM analyses. This indicates that 2D plane strain investigations of the failure process are conservative with respect to the probability of initial and retrogressive failures.

For the example problem considered, secondary failures on the sides of the original failure were more likely than retrogressive failure away from the crest. This failure pattern is beneficial for dyke slope failures, since lateral spreading of the failure will not (directly) trigger flooding. An increase in the degree of anisotropy increases the likelihood of retrogressive failures and tends to increase the width of the failures, while a smaller degree of anisotropy results in a more chaotic failure process where many small zones can become unstable. For isotropic spatial variability (and small values of the scale of fluctuation) the results approximate to the deterministic outcome due to the averaging of properties over potential failure planes. The results for degrees of anisotropy larger than 5 are similar. A small depth trend increases the resistance against initial and retrogressive failure as the strength at the bottom of the slope increases. However, a larger depth trend causes a decrease in the ultimate foundation load and a greater tendency for retrogressive behaviour, due to the increased likelihood of failures at multiple depths. For a large depth trend, secondary failures along (approximately) circular failure surfaces become less likely; instead, weak material tends to flow into an expanding failure zone.

## Data Availability

The analyses can be reproduced based on the information provided here. The data sets generated during the study are available from the corresponding author on reasonable request.
